# Mediators of Inflammation in Obesity and Its Comorbidities

**DOI:** 10.1155/2010/239126

**Published:** 2010-09-21

**Authors:** Oreste Gualillo

**Affiliations:** Santiago University Clinical Hospital, Neuro Endocrine Interactions in Rheumatology and Inflammatory Diseases Laboratory, Research Laboratory 9, Building C, Travesía da Choupana s/n, 15706 Santiago de Compostela, Spain

In the last decades we have seen a rapid expansion of the proportion of obese individuals worldwide. Actually obesity has gradually but also rather considerably revealed as a whole medical problem with menacing implications for the health of our society. Indeed, obesity is already of epidemic proportions in the U.S. and in many other parts of the world and it is a worrying problem also in developing countries. According to the last data of World Health Organization, more than 1 billion people are overweight worldwide, where more than 300 million fulfil obesity criteria [1]. Obesity is accompanied by a plethora of other relevant diseases including cardiovascular, cerebrovascular, and liver diseases, type 2 diabetes, and dyslipidemias that are affecting also youngest age groups of population. Obesity has been recently recognized also as a relevant contributing risk factor for certain types of cancer [2]. 

Emerging literature suggests that inflammation, as evaluated by high inflammatory cytokines levels and other inflammatory markers, may represent basically a cause and consequence of obesity and its comorbidities [3]. We have to bear in mind that obesity is a consequence of millions of years of evolution during which the ability to store efficiently fat in long periods of fluctuation of food availability, alternated by famines, granted capacity to survive, reproduce, and maintain survival of offspring. In the last 50 years, characterized by an excess of food availability, this adaptive skill shifted to maladaptive increasing the propensity to obesity and its comorbidities above mentioned. Several disorders that represent the major sources of morbidity and mortality in the present world such as type 2 diabetes, cardiovascular disease, and cancer are known to be associated with obesity and have recently been reconsidered as inflammatory diseases. For instance, the inflammation associated with the accumulation of intra-abdominal fat is associated with progressive resistance to the effects of insulin, ultimately leading to type 2 diabetes [4]. Anyway, the causes for the activation of the inflammatory response in obesity and its comorbidities are undoubtedly complex and we are in the beginning of the road to cover. Indeed, increasing evidence (most of them reported in this special issue) suggests a possible causal link between adiposity, particularly visceral obesity and inflammation. Obesity leads to increases in inflammatory cytokines and adipocytokines and changes in related molecules such as leptin and adiponectin, which may contribute to the development of multiple disturbs in predisposed individuals. However, in most cases the relationship appears to be bi-directional, in that prior comorbidities seem to increase and perpetuate the proinflammatory status associated to adiposity. Clearly, more research is needed to examine the complex interplay between inflammation and adiposity, an effect that is also in part to consequences of low physical activity. However, the interactions between these systems offer unique opportunities for targeted treatment and prevention strategies. 

For instance, there is a pressing need to investigate strategies to disrupt inflammatory signalling in persons with obesity (morbid or not) who show indication of a proinflammatory state (e.g., those with elevated CRP). Research on approaches that alter diet, increasing the intake of particular micronutrients, or, of course, reducing weight, is also needed [5]. 

This special issue has focused on adiposity and inflammation as one of the main contributing factors for obesity-associated morbidities. 

However, several salient facts must be noted. Obesity and comorbidities are complex and multifaceted diseases with too many contributing factors. So, here we intended to suggest that inflammation and adiposity contribute strongly in all or even most affected people. The prevention of obesity would significantly reduce the burden of comorbid diseases. Indeed, by decreasing prevalence of obesity, other obesity-related conditions can be reduced to a greater extent and this will help in cutting down the health care budget to a great extent (in times of economic crisis as such we are living everywhere, this should be mandatory considered).

One year ago approximately, I answered to a call of our Editor in Chief, Professor Freek Zijlstra, suggesting a special issue on mediators of inflammation in obesity and its comorbidities, and he has indeed selected a worthy topic. 

In this issue of Mediators of Inflammation, we are pleased to present to the reader a series of special features written by great authorities in the field. In this special issue, the reader will find several articles (more than 25) written by experts on epidemiology, mechanisms and molecules, regulation of body weight, concomitants of obesity, and therapeutic approaches to obesity. 

I would like to thank all contributors and reviewers and I am personally grateful for your support to this special issue as there can be no growth or improvement without your participation. 

Finally, a special thank is also due to the Associate Editors of this special issue Giamila Fantuzzi, Gema Fruhbeck, Giuseppe Matarese, and Paul Trayhurn for their hard work, commitment, and support. 

*Oreste Gualillo* *Oreste Gualillo*
